# Immunomodulatory Effect of Mesenchymal Stem Cells on B Cells

**DOI:** 10.3389/fimmu.2012.00212

**Published:** 2012-07-20

**Authors:** Marcella Franquesa, M. J. Hoogduijn, O. Bestard, J. M. Grinyó

**Affiliations:** ^1^Department of Internal Medicine, Erasmus Medical CenterRotterdam, Netherlands; ^2^Department of Nephrology, Hospital de Bellvitge UB-IDIBELLBarcelona, Spain

**Keywords:** MSC, B cells, humoral rejection, chronic allograft rejection, immunomodulation

## Abstract

The research on T cell immunosuppression therapies has attracted most of the attention in clinical transplantation. However, B cells and humoral immune responses are increasingly acknowledged as crucial mediators of chronic allograft rejection. Indeed, humoral immune responses can lead to renal allograft rejection even in patients whose cell-mediated immune responses are well controlled. On the other hand, newly studied B cell subsets with regulatory effects have been linked to tolerance achievement in transplantation. Better understanding of the regulatory and effector B cell responses may therefore lead to new therapeutic approaches. Mesenchymal stem cells (MSC) are arising as a potent therapeutic tool in transplantation due to their regenerative and immunomodulatory properties. The research on MSCs has mainly focused on their effects on T cells and although data regarding the modulatory effects of MSCs on alloantigen-specific humoral response in humans is scarce, it has been demonstrated that MSCs significantly affect B cell functioning. In the present review we will analyze and discuss the results in this field.

## Introduction

B cells are a major cell type involved in adaptive immune responses, specialized in antigen presentation and antibody production. The balance between the different B cell subsets has been identified as an important factor for graft outcome. On one hand, effector B cells generate humoral rejection and pre-formed donor-specific antibodies (DSA) against human leukocyte antigen (HLA)-I or HLA-II that have been correlated to worst graft outcome. On the other hand, pro-tolerogenic B cell subsets have been identified. An increase in immature transitional and naïve B cells has been related to tolerance (Liu et al., 2007) and increased B cell numbers and a differential expression of B cell-related genes were observed in the peripheral blood of a small cohort of tolerant kidney and liver transplant patients compared to stable patients under immunosuppression or to healthy controls (Newell et al., 2010; Sagoo et al., 2010).

Mesenchymal stem cells are multipotent stromal cells localized in virtually every tissue. They are characterized by their adherence to plastic, the expression of surface markers as CD73, CD90, and CD105 among others and the lack of expression of typical hematopoietic markers as CD45 and CD11b (Roemeling-van Rhijn et al., 2012). They also show differentiation potential into different cell lineages under controlled culture conditions. MSCs have been considered as naturally immunoprivileged cells due to low expression of HLA and co-stimulatory molecules in unstimulated conditions and although it is now well-known that under inflammatory stimulation they can express both HLA-I and HLA-II it is also known that under this condition they exert more potent immunosuppressive actions (Crop et al., 2010).

The effect of MSCs on effector and regulatory T cells has been widely studied (Duffy et al., 2011a) and there is also evidence for a suppressive role of MSCs on natural killer (NK) cells (Spaggiari et al., 2008), inhibition of dendritic cells (DCs) maturation (Spaggiari et al., 2009), and alternative activation of macrophages leading to an anti-inflammatory phenotype (Francois et al., 2012). The interaction between MSCs and B cells is gaining interest but data is still scarce and controversial. Here we review the available data on the immunomodulatory actions of MSCs on B cells.

## B Cells in Transplantation

T cell-mediated rejection is together with antibody-mediated rejection the main cause of graft loss. Although research on T cell immunosuppressive therapies has efficiently improved the incidence of acute cellular rejection, long-term allograft survival remains challenged by chronic rejection. Activated B cells have been found to play a significant role on long-term allograft function. Their ability to present antigen to T cells via the indirect pathway and the generation of DSAs are emerging as the major mediators of allograft rejection. Pre-existing DSAs in the allograft recipient mediate hyperacute and acute-antibody-mediated rejection while the presence of *de novo* DSAs (specific for HLA and non-HLA) in recipients compromises long-term allograft survival (Redfield et al., 2011). Furthermore, it has been observed that CD8 and CD4 T cell memory is impaired when the antigen presenting function of B cells is absent (Ng et al., 2010). This finding would support the idea of a beneficial effect of B cell depletion at the time of transplantation to impair T cell mediated alloresponses.

However, there is increasing evidence for a tolerogenic role of specific B cell subsets. Naïve B cells have been shown to stimulate the development of regulatory T cells by antigen presentation to naïve T cells (Reichardt et al., 2007). And more recently, increased expression levels of B cell genes were found in peripheral blood of kidney transplant patients that spontaneously became tolerant (Newell et al., 2010; Sagoo et al., 2010). The three main genes with predictive value for discerning tolerant from non-tolerant (*IGKV4-1*, *IGLL1*, and *IGKV1D-13*) are expressed by transitional B cells, which are considered to be tolerogenic. Moreover, there is evidence of a subset of B cells with anti-inflammatory properties and the ability to secrete IL10, which down-regulation is known to be involved in the development of autoimmune diseases (Mauri and Bosma, 2011) and in solid organ transplantation there is preliminary evidence for their presence in immunosuppressive free kidney transplant patients (Le Texier et al., 2011).

The use of B cell directed monoclonal antibodies (anti-CD20, Rituximab), antibody depleting strategies (plasmapheresis), plasma cell depleting agents (anti-proteasome, Bortezomib), or complement-inhibitor agents (Eculizumab) have been reported to be efficient in promoting graft survival (Rocha et al., 2003; Tyden et al., 2009; Walsh et al., 2012). It is however still controversial which approach is the best to avoid humoral rejection without compromising regulatory mechanisms.

## Immunomodulatory Effect of MSCs in Transplantation

T cells, as key initiators and mediators of transplant rejection, have been the main target to prove the immunomodulatory potential of MSCs. Multiple studies have demonstrated that MSCs inhibit effector T cell proliferation and cytokine release through mechanisms that are cell-contact-dependent (PD-L1, Augello et al., 2005; B7-H4, Xue et al., 2010; ICAM1; VCAM1, Ren et al., 2010) and contact-independent (IDO, Ge et al., 2010; PGE_2_, Najar et al., 2010; Duffy et al., 2011b; HLA-G, Selmani et al., 2009; TGFβ, Liu et al., 2012; galectins, Sioud, 2011). *In vivo* models have shown that MSCs have an indirect effect on T cell activation by inhibition of maturation of DCs. Injected MSCs prevent DCs maturation (Spaggiari et al., 2009) and their migration to lymph nodes by down-regulating CCR7 expression, thus inhibiting T cell priming (Chiesa et al., 2011). MSC-exposed DCs have also the ability to promote Tregs induction (Ge et al., 2009). MSCs posses also the ability to induce Tregs directly via the production of TGFβ, PGE_2_ together with cell-contact as key factors. *In vivo*, FoxP3^+^ Treg generation has been associated with IDO expression by MSC (English et al., 2009). This factor is produced by MSCs under IFNγ conditioning (Croitoru-Lamoury et al., 2011) and is essential to achieve allograft tolerance in an experimental kidney transplantation model (Ge et al., 2010). It appears that MSCs under inflammatory conditions act as super-regulators on T cells inhibiting the effector responses and enhancing the regulation inducing Tregs.

Of note, these actions are not only relegated to the experimental and *in vitro* setting as the applicability of injected MSCs as induction therapy in human kidney transplantation has been recently proved. Injection of autologous MSCs at the moment of transplantation and 2 weeks post-transplantation resulted in lower incidence of acute rejection, decreased risk of opportunistic infection and better estimated renal function at 1 year compared to anti-IL2 receptor antibody (Basiliximab) induction therapy (Tan et al., 2012).

## Effect of MSCs on B Cells *In vitro*

To the moment, the few published papers studying the effect of MSCs on B cells proliferation, differentiation, and function show disparity in their approaches and results. The different results among the groups might be explained by the different starting B cell population (purity and isolation method) and the stimuli used to trigger B cell differentiation and proliferation. MSC: B cell ratio is also an important point, as the most effective ratios used are very high and it is hardly observed a dose dilution effect, contrarily to what happens with the immunosuppressive effect of MSCs on T cells (Hoogduijn et al., 2008).

### Cell source and isolation method

If we refer to *in vitro* data (Table 1), the main starting difference of those studies is the B cell isolation method. On one hand, some authors decided for a more “physiological” model by using a B cell enriched system in which we can still find T helper cells (in different proportion depending on the depleting technique and the source used) and other mononuclear cells found in peripheral blood or spleen (Rasmusson et al., 2007; Comoli et al., 2008). On the other hand, some authors use CD19 positive selection to start with a pure B cell population (Corcione et al., 2006; Tabera et al., 2008; Traggiai et al., 2008), or a CD43 depleted population to have an isolated “untouched” non-activated B cell population to start with (Asari et al., 2009; Schena et al., 2010). The purity of the starting population and the stimuli used to trigger B cell proliferation and differentiation are key factors in determining the effect of MSCs on B cells.

**Table 1:** **Effect of MSCs on B cells *in vitro***.

Species and model	B cell isolation	B: MSC ratio	B cell stimuli	Effect of MSCs	Reference
**MICE**					
Mice C57Bl/10 and Balb/C	Spleen B cell isolation kit	1:1	PWM	Inhibition of B cell proliferation. PD-1/PD-1L/PD-2L pathway is involved.	Augello et al. (2005)
Mouse B6 and CCR2−	Spleen sorted CD19−CD138^+^ plasma cells	1:1	rOVA	Inhibition of Ig production by a cleaved form of CCL2 secreted by MSCs.	Rafei et al. (2008)
Mouse C57Bl/g	Spleen CD43 depletion	2:1 5:1 10:1	LPS T cell dependent/independent *in vivo*	Inhibition of Plasma cells (Blimp-1+) induced by LPS. Suppression of B cell proliferation but do not induce plasma cell apoptosis. B cell differentiation inhibition is cell-contact-independent (also not CCL2, IL10, TGFβ, or IDO).	Asari et al. (2009)
Mouse NZBxNZW F1	Spleen CD43 depletion and sorted marginal vs. follicular zone	1:1 3:1 9:1	CpG + CD40L + anti-IgM + IL2	Inhibition of proliferation and differentiation in the presence of IFNγ of BCR stimulated naive B cells. This effect is IDO independent and cell-contact-dependent and not related to apoptosis. Inhibition of phosphorylation of 3 main pathways downstream de BCR and PD-1/PD-L1 upstream the BCR.	Schena et al. (2010)
Mouse NZBxNZW F1	Spleen, BM, kidney CD138^+^ plasma cells isolation	1:1 1:5	OVA	Coculture MSCs increase survival and function of plasma cells leading to increased IgG production.	Youd et al. (2010)
**HUMANS**					
Human healthy volunteer	PB T cell depleted + CD19^+^ positive selection MACS	1:1 1:2	CpG + rCD40L + anti-Ig + IL2 + IL4 ± IL10	Inhibition of proliferation (not apoptosis) by arrest of cell cycle G0/G1. Mediated by soluble factors. Inhibition of IgG, IgA, IgM secretion. Inhibition of homig molecules CXCR4, CXCR5, CCR7, and chemotaxis to CXCL12, CXCL13.	Corcione et al. (2006)
Human healthy volunteer	Spleen or PB enriched B cell population MACS	10:1	LPS/CMV/VZV	Increase IgG producing cells in coculture with MNCs or B cells. The effect on enriched B cells is cell-contact-dependent while is mediated by soluble factors in MNCs. Under strong stimulation MSC reduce Ig production, under low stimulation increases Ig production.	Rasmusson et al. (2007)
Human healthy volunteer and highly sensitized patients	PB partial depletion CD4 and full depletion CD8 MACS	4:1 20:1	MLC ± CD40 agonist + IL10	MSCs inhibit IgG, IgA, IgM production induced in MLC (different ratios and allogeneic or syngeneic MSCs have same effect). Sensitized patients allo-sera induce ADCC but supernatant of MLC + MSC do not induce ADCC. In the presence of agonist CD40 + IL10, MSCs have no effect on Ig reduction and in transwell the effect is not lost.	Comoli et al. (2008)
Human healthy volunteer	Buffy Coat CD19^+^ and CD3^+^ selection MACS	5:1 10:1	CpG + anti-Ig ± CD40L + IL4	Promotion of B cell proliferation and viability but under highly proliferative conditions, MSCs arrest B cell cycle in G0/G1. Inhibit Plasma cells induced by pDCs mediated by ERK 1/2 and p38 phosphorylation.	Tabera et al. (2008)
Human healthy and SLE	PB CD19^+^ selection MACS + subsets sorting	1:1	CpG + IL2 ± CD40L + anti-Ig	Induction of survival and proliferation of transitional, naïve, IgM memory, and switch memory subsets with/out stimulation. Up-regulation of CD38 and IGM but naïve B cells do not increase IgA and IgG. Cell-contact-dependent effect. Enhancement of survival of SLE patient B cell subsets, increase CD38 expression and IgM and IgG secretion.	Traggiai et al. (2008)

*Summary of the published works on the effect of MSCs on B cells *in vitro*. In all cases the source of MSCs is bone marrow. PWM, Pokeweed mitogen; OVA, ovalbumin; LPS, lipopolysaccharide; BM, bone marrow; PB, peripheral blood; MACS, magnetic cell sorting technology; CMV, cytomegalovirus; VZV, varicella zoster virus; MNCs, mononuclear cells; MLC, mixed lymphocyte culture*.

Of note, the source of MCS used in the various studies is bone marrow and the use of allogeneic or autologous MSCs does not seem to affect the interaction between MSC and B cells (Comoli et al., 2008).

The first key study to understand the role of MSC on B cells, on a non-purified starting population, was performed by Comoli et al. (2008). The exposure of enriched B cell populations to irradiated third party PBMCs led to an increase in immunoglobulin (Ig) production that was abrogated by the addition of MSCs. Interestingly the effect exerted by MSCs was abolished by the addition of anti-CD40 and IL10 indicating that MSCs suppression of Ig production was produced by T help suppression rather than by a direct effect on B cells. This is in tune with Rasmusson et al. (2007) who showed that under strong stimulation of mononuclear cell fraction (non-purified B cells), MSCs inhibited the Ig secretion. However, the same cells without or under mild polyclonal stimulation increased their IgG production in the presence of MSCs (Rasmusson et al., 2007).

However, when the effect of MSCs is studied on purified B cells (or B cell subsets) the effect is diverse depending on the stimuli used to induce proliferation and/or differentiation.

### Cell stimulation

The activation of naïve B cells requires three signals: B cell receptor (BCR) activation (via anti-Ig), T cell co-stimulatory help (via CD40/CD40L), and appropriate cytokines or toll-like receptor (TLR) activation (microbial products, CpG, dsRNA), while memory B cells can be activated in the absence of BCR stimulation and triggered via stimulation of TLR or bystander T cell help only (Lanzavecchia et al., 2006). MSC may have a role in modulating some of these B cell activating pathways.

Among the studies that isolated pure B cells and exposed them to different stimuli to analyze the effect of MSCs we find diverse results. Although MSC were shown to increase the viability of B cells (Tabera et al., 2008), they arrest them in G_0_/G_1_ (Corcione et al., 2006; Tabera et al., 2008) and inhibit their differentiation into plasma cells and subsequent Ig formation. This effect has been shown to be cell-contact-independent (Asari et al., 2009) or indirect through inhibition of pDCs induced B cell maturation (Tabera et al., 2008). Contrarily, some authors (Augello et al., 2005; Schena et al., 2010) found PD-1/PD-L1 interaction and the inhibition of pathways downstream the BCR (Schena et al., 2010) to be responsible for B cell inhibition by MSC. However, Schena et al. (2010) observed that pre-exposure of MSCs to IFNγ was mandatory for their suppressive effect on B cells, similar to their effect on T cells (Crop et al., 2010). These studies on isolated B cells were performed in the presence of stimuli targeting the three signals for B cell activation, suggesting a role of MSCs directly on B cells besides their effect on T helper cells (contrarily to what was observed in studies using mixed starting population).

In contrast to activated B cells, isolated naïve, transitional, and memory B cell subsets exposed to MSCs increased their survival and proliferation (Traggiai et al., 2008). MSCs synergize with TLR stimuli and IL2, with or without T cell help (CD40L or anti-CD40) and BCR mediated stimulation by inducing proliferation and differentiation into plasma cells. This effect was shown to be contact-dependent although some of the factors released by MSCs are important to modulate this effect.

In this setting, the effect of the stimuli on MSCs should be also taken into account. It has been proved that MSCs express TLRs (DelaRosa and Lombardo, 2010), and their activation promote mainly a different cytokine secretion. MSC stimulated with CpG (one of the main stimuli used in B cell activation that acts through TLR9) produce IL6. This cytokine stimulates B cell proliferation (Friederichs et al., 2001) and could be an explanation for the MSC induction of naïve B cell proliferation under TLR9 stimulation in the absence of BCR triggering (Traggiai et al., 2008).

All these studies give a hint on a potential dual effect of MSCs on B cells. While in the enriched system the effect of MSCs on B cells appear to be by-passed by their immunosuppressive action on T cells, in an activated pure B cell population MSCs efficiently arrest or increase the proliferation depending on the potency of the stimuli on B cells but also on MSCs. Both cell-contact-dependent and independent factors are involved.

### Effect of MSCs on plasma cells

Mesenchymal stem cells inhibit plasma cell formation induced by allostimulation (Comoli et al., 2008), by LPS (Asari et al., 2009) or by plasmatic DCs (Tabera et al., 2008) and subsequent Ig production (Corcione et al., 2006; Rasmusson et al., 2007). The mechanisms of action described to be involved are cell-contact-independent (alternatively cleaved CCL2 Rafei et al., 2008) or dependent (PD-1/PD-L1 interaction, Schena et al., 2010).

However, we also find some disparity in the results obtained *in vitro*, as some authors observed and increased differentiation into plasma cells with increased Ig production (Traggiai et al., 2008) along with a better survival and function (Youd et al., 2010). This observation is reflected in *in vivo* systemic lupus erythomatosus (SLE) models treated with MSCs.

## Effect of MSCs on B Cells *In vivo*

Similar to the controversial *in vitro* effects of MSC on B cells, there are contradictory reports on the effects of MSC on B cells in animal models.

Different groups have approached the treatment of a SLE model with MSCs. A single injection of human BM-MSCs combined with cyclophosphamide (CTX) increased survival, decreased proteinuria, and reduced the levels of circulating anti-dsDNA IgG in a MRL/Lpr mice model (Zhou et al., 2008), and similar results were obtained in NZBxNZW F1 mice injected preventively with adipose tissue MSCs every 2 weeks for 54 weeks although this protective effect was lost when the animals were treated after the onset of the disease (Choi et al., 2012). This late treatment does not prevent from developing anti-dsDNA IgG or proteinuria, neither increases the survival of the treated animals but it decreases lymphocytic infiltration, glomerular proliferation, and immune complex deposition (Schena et al., 2010). Contrarily, the use of mouse allogeneic MSCs in this model, increases serum anti-dsDNA antibodies and the glomerular deposition of IgG, along with higher interstitial fibrosis and inflammation and protein casts in the kidney when compared to CTX treatment (Youd et al., 2010). Of note the numbers of IgG^+^ plasma cells in the bone marrow of MSC treated mice are also increased (Table 2).

**Table 2:** **Effect of MSCs on B cells *in vivo***.

Model	Species	MSC source	MSC dose	Effect of MSCs	Reference
SLE	Mouse female MRL/Lpr	BM human	1 × 10^6^/mice	MSCs alone or combined with Cyclophosphamide (CTX) reduce serum creatinine levels and C3 deposition compared to CTX alone. CTX + MSC reduce circulating dsDNA antibodies.	Zhou et al. (2008)
Heart allograft	Mouse C57BL/6 BALB/c C3H	BM	1 × 10^6^/mice	Inhibition of intragraft and circulating alloreactive antibody levels. In combination with rapamycin induce tolerance.	Ge et al. (2009)
SLE	Mouse C57Bl/g	BM conditioned medium	Conditioned medium	Suppression of antigen specific IgM and IgG1 secretion in immunized mice with T cell-dependent and -independent effect	Asari et al. (2009)
SLE	Mouse NZBxNZW F1	BM C57BL/6J mice	3 Injections 1.25 × 10^6^	Injections of MSC in SLE mice has no effect on IgG dsDNA, proteinuria and survival, but improves glomerular proliferation, lymphocytic infiltration, and IgG immune complex deposition.	Schena et al. (2010)
SLE	Mouse NZBxNZW F1	BM Allogeneic Balb/C	1 × 10^6^ Bi-weekly for 18 or 7 weeks	MSC enhance autoantibody production, pathology and proteinuria.	Youd et al. (2010)
SLE	Mouse NZBxNZW F1	AT human	28 Injections 5 × 10^5^	Higher survival, improvement of histologic, and serologic abnormalities and immunologic function and decreased proteinuria. Anti-dsDNA antibodies and BUN decreased. GM-CSF, IL4, and IL10 increase. Increase of Tregs proportion. Early injections have best results than late treatment.	Choi et al. (2012)

*Summary of the published works on the effect of MSCs on B cells *in vivo*. BM, bone marrow; SLE, systemic lupus erythematosus; AT, adipose tissue*.

In the transplantation setting, we (Franquesa et al., 2012) and others (Ge et al., 2009) have demonstrated that MSCs injection can significantly reduce levels of allospecific circulating antibodies and intragraft allospecific IgG deposits (Ge et al., 2009) leading to long-term graft acceptance.

## Induction of B Cell Responses by Allo-MSC

Despite the low immunogenicity that MSCs are supposed to exert (low HLA class I and negative for HLA class II in unstimulated conditions), there is evidence that MSC may be capable of inducing an adaptive immune response (Nauta et al., 2006; Sbano et al., 2008). Therefore it is still a matter of debate whether allogeneic MSCs exert a humoral response in the recipient.

In rats a single injection of allogeneic MSC (1 × 10^6^ cells/animals) induced substantial alloantibody production (IgG1, IgG2) in contrast to syngeneic cells injection in an immunocompetent host (Schu et al., 2011). Also immunocompetent non-human primates (baboons) injected with two doses of allogeneic MSCs (5 × 10^6^ cells/kg body weight) developed alloantibodies (Beggs et al., 2006). Contrarily, in a clinical study with 12 patients which were treated with MSCs (0.8–2.0 × 10^6^ cells/kg body weight) after hematopoietic stem cell (HSC) transplantation, none of them developed anti-MSC antibodies (Sundin et al., 2007). And our own experience with a single injection of third party MSCs (0.5 × 10^6^/300 g body weight) in a rat kidney transplantation model is that the injected animals do not develop specific anti-MSCs antibodies while they do increase antibody levels against the third party when they are injected with the full fraction of bone marrow mononuclear cells (Franquesa et al., 2012).

Those studies reflect some disparity of humoral response directed against the injected MSCs that could be explained by the source of MSCs (allo- vs. syngeneic), the number of injected cells, the number of injections, the route of administration or concurrent immunosuppression used. More *in vivo* studies need to be done to develop safe long-term protocols for the clinical setting.

## Conclusion

The role of B cells in transplantation is multifaceted due to the opposed roles of different B cell subsets in tolerance and rejection. This enlightens the need for more refined immunosuppressive regimens to treat humoral rejection without compromising the effect of the pro-tolerogenic B cell subsets, namely transitional and regulatory B cells.

Mesenchymal stem cells have proven immunomodulatory properties, suppressing inflammatory cell (effector T cells, DCs, inflammatory macrophages) functions, and differentiation and increasing or synergizing with regulatory cells such as Tregs.

Their effect on B cells has been scarcely studied and although the results obtained are contradictory so far, it seems clear there is a close interaction between MSC and B cells. It appears that this interaction occurs partly through the modulation of T cell help by MSCs, but also in the absence of helper cells MSCs can inhibit activated B cells. The study of this threesome relation is of special interest in the transplantation setting. Another interesting point that remains to be studied is the potential of MSCs to induce pro-tolerogenic B cell subsets that have themselves proved immunomodulatory properties.

The potential of MSCs in B cell immunomodulation appears to be promising and not fully understood. The advent of new and well designed studies can give important insights to fully picture the therapeutic role of MSCs in B cell mediated rejection.

## Conflict of Interest Statement

The authors declare that the research was conducted in the absence of any commercial or financial relationships that could be construed as a potential conflict of interest.
